# Understanding the Burden and Management of Urinary Tract Infections in Women

**DOI:** 10.3390/diseases13020059

**Published:** 2025-02-15

**Authors:** Baiken Baimakhanova, Amankeldi Sadanov, Lyudmila Trenozhnikova, Assya Balgimbaeva, Gul Baimakhanova, Saltanat Orasymbet, Diana Tleubekova, Alma Amangeldi, Zere Turlybaeva, Zhanar Nurgaliyeva, Roza Seisebayeva, Zhanat Kozhekenova, Saltanat Sairankyzy, Zhanserik Shynykul, Sandugash Yerkenova, Aknur Turgumbayeva

**Affiliations:** 1 LLP “Research and Production Center for Microbiology and Virology”, 105 Bogenbay Batyr Str., Almaty 050010, Kazakhstan; bbbayken@mail.ru (B.B.); a.sadanov1951@gmail.com (A.S.); barahtian@yandex.ru (L.T.); imv_rk@list.ru (A.B.); bgulb@mail.ru (G.B.); s_orazymbet@inbox.ru (S.O.); dianatleubekova1@gmail.com (D.T.); almashka91@mail.ru (A.A.); tzj2009@yandex.kz (Z.T.); 2 Department of Outpatient Pediatrics, School of Pediatrics, S.D. Asfendiyarov Kazakh National Medical University, 96 Tolebi Str., Almaty 050010, Kazakhstan; nurgaliyeva.z@kaznmu.kz (Z.N.); seisebaeva_68@nail.ru (R.S.); 3 Department of Public Health, S.D. Asfendiyarov Kazakh National Medical University, 96 Tolebi Str., Almaty 050010, Kazakhstan; kozhekenova@mail.ru; 4 Department of Propaedeutics of Childhood Diseases, School of Pediatrics, S.D. Asfendiyarov Kazakh National Medical University, 96 Tolebi Str., Almaty 050010, Kazakhstan; sairankyzy.s@kaznmu.kz; 5 Higher School of Medicine, Al-Farabi Kazakh National University, Almaty 050040, Kazakhstan; shynykul.zhanserik@med-kaznu.com

**Keywords:** urinary tract infections, pregnancy, pelvic organ prolapse, management, postmenopausal women, multidrug resistance, diagnosis, uUTI, cUTI

## Abstract

Urinary tract infections (UTIs) represent a prevalent health concern among the female population, with anatomical and physiological determinants such as a shorter urethra and its proximity to the rectum augmenting vulnerability. The presence of Escherichia coli and various other pathogens plays a significant role in the etiology of these infections, which can be aggravated by sexual intercourse and disturbances to the vaginal microbiome. The physiological alterations associated with pregnancy further elevate the likelihood of UTIs, with untreated cases potentially leading to severe complications such as pyelonephritis, preterm labor, and stillbirth. Furthermore, postmenopausal women encounter an augmented risk of UTIs attributable to estrogen deficiency and vaginal atrophy, as well as conditions including pelvic organ prolapse (POP) and urinary incontinence (UI), which hinder optimal bladder functionality. The aforementioned factors, in conjunction with the rising prevalence of cesarean deliveries and catheterization, complicate the management of UTIs. While precise diagnosis is paramount, it remains a formidable challenge, notwithstanding advancements in molecular diagnostic techniques. Management strategies encompass antibiotic-sparing therapies; however, the increasing incidence of multidrug resistance represents an alarming trend. Diverse guidelines from various medical specialties endeavor to standardize treatment approaches, yet significant inconsistencies continue to exist. This study systematically appraises the extant guidelines, evaluating the quality of evidence while identifying areas of agreement and discord to supply practitioners with effective strategies for UTI management.

## 1. Introduction

Urinary tract infections (UTIs) are more prevalent in women due to their shorter urethra and its proximity to the rectum, which facilitates bacterial entry into the urinary tract [1]. Additionally, infections often affect the vulvar vestibule, a region particularly prone to inflammation and infection, especially in cases of vulvar vestibulitis and vaginitis. In such instances, factors such as sexual intercourse and excessive use of intimate hygiene products, which can disrupt the natural vaginal microbiome, are frequently implicated [2].

The close anatomical relationship between the anus and the reproductive organs, as well as the distal segments of the urinary tract in females, facilitate the opportunistic colonization by microorganisms such as *Escherichia coli*, *Klebsiella pneumoniae*, *Enterococcus faecalis*, *Proteus mirabilis*, *Staphylococcus saprophyticus*, *Candida albicans*, *Gardnerella vaginalis*, and various species of *Streptococcus* [3,4,5]. These microorganisms possess the capability to translocate from the gastrointestinal system to the urethra, potentially resulting in infectious conditions. Consequently, this specific anatomical characteristic, when coupled with additional physiological susceptibilities, significantly heightens the probability of infections affecting the urinary tract and reproductive organs in females [6].

Pregnancy and the perinatal period signify pivotal stages in a woman’s life, frequently characterized by a heightened prevalence of UTIs. Symptomatic UTIs are documented in approximately 1% to 2% of all pregnancies, whereas asymptomatic bacteriuria impacts 2% to 13% of pregnant individuals [7]. Physiological and hormonal alterations during gestation, including ureteral dilation and urinary stasis, enhance this susceptibility. If not addressed, UTIs occurring during pregnancy can result in severe adverse outcomes, such as pyelonephritis, infants with low birth weight, premature labor, and stillbirth. Consequently, the prompt identification and intervention of both symptomatic UTIs and asymptomatic bacteriuria are of paramount importance [8,9].

An additional risk factor for UTIs is the rising frequency of cesarean deliveries and perioperative catheterization procedures. Postmenopausal women are also confronted with an elevated risk of UTIs due to diminishing estrogen levels, which lead to atrophy of the vaginal epithelium and a decline in lactic acid bacteria, particularly *E. coli*, which may colonize the vaginal area and subsequently disseminate to the urinary tract [10]. Moreover, pelvic organ prolapse (POP) and urinary incontinence (UI) are critical factors contributing to recurrent UTIs among women. Both conditions result in urinary retention and incomplete bladder evacuation, thus fostering an environment favorable for bacterial proliferation and infection [11]. The interplay between POP, UI, and UTIs is complex. Women afflicted with these conditions frequently report a higher incidence of UTIs attributable to impaired bladder functionality. In addition, postmenopausal status, along with childbirth, are significant variables that predispose women to both POP and UI, thereby further complicating urinary health [12]. Some investigations have demonstrated a correlation between the intensity of POP and lower urinary tract symptoms (LUTS), inclusive of elevated UTI incidence. However, it remains imperative to acknowledge that not all women with POP or UI will encounter recurrent UTIs, as individual health determinants and effective management approaches are critical in influencing clinical outcomes [13,14,15,16].

Accurate diagnosis and effective management of UTIs are essential due to their significant health and economic burden on both individuals and society. While urine microscopy and culture have long been the diagnostic ‘gold standard,’ their limitations in sensitivity and specificity are well documented [17]. Advances in molecular diagnostics, such as whole genome sequencing and metagenomic sequencing, offer promising improvements in diagnostic accuracy. However, these techniques are not yet validated for routine clinical practice [18]. While antibiotic treatment for asymptomatic bacteriuria in individuals without specific risk factors is discouraged, inconsistent and widespread use persists in both symptomatic and asymptomatic populations, raising concerns about the emergence of multidrug-resistant pathogens [19]. As a result, interest in antibiotic-sparing approaches, including vaginal estrogen for postmenopausal women, cranberry supplements, acupuncture, and immunoactive prophylaxis, has grown, although recommendations remain variable [20,21].

Given the cross-disciplinary nature of UTI management, multiple medical specialties, including general practice, internal medicine, geriatric medicine, infectious diseases, gynecology, and urology, are involved in care delivery [22,23]. Numerous guidelines have been formulated and disseminated by these professional groups to facilitate the standardization of evidence-based treatment protocols. Nonetheless, considerable uncertainty persists, alongside variability in the robustness and substance of these recommendations [24,25,26,27]. Consequently, in order to mitigate ambiguity for practitioners managing patients with UTIs, the objective of this investigation was to systematically evaluate the existing guidelines based on the quality and rigor of evidence underpinning their recommendations and to identify areas of both agreement and divergence.

## 2. Materials and Methods

In March 2024, scholarly databases such as PubMed and Google Scholar, alongside the official platforms of prominent organizations specializing in urology, gynecology, infectious diseases, and general practice, were scrutinized for clinical guidelines pertaining to UTIs. A comprehensive evaluation resulted in the selection of five distinct guidelines, which encompass those issued by the European Association of Urology (EAU), the Swiss Society of Gynecology and Obstetrics (SSGO), the German Association of Scientific Medical Societies (AWMF), the American Urological Association (AUA) in conjunction with the Canadian Urological Association (CUA)/the Society of Urodynamics, Female Pelvic Medicine & Urogenital Reconstruction (SUFU), and the Kazakhstan Association on Sexual and Reproductive Health (KASRH). To ensure the relevance and quality of the selected guidelines, we applied specific inclusion criteria, such as guidelines published in English within the last 30 years with a focus on the clinical management of UTIs, and those that provided evidence-based recommendations. The exclusion criteria included outdated guidelines (published more than 30 years ago), studies not peer-reviewed, or guidelines not specifically addressing the clinical management of UTIs, as well as those that were focused solely on theoretical aspects or lacked clinical applicability. After conducting an initial search, we retrieved 345 articles and screened them based on these criteria. A final total of 123 articles were included in the review, ensuring a comprehensive yet focused selection of relevant clinical guidelines.

## 3. Categories and Diagnosis of UTI

**Figure 1:**
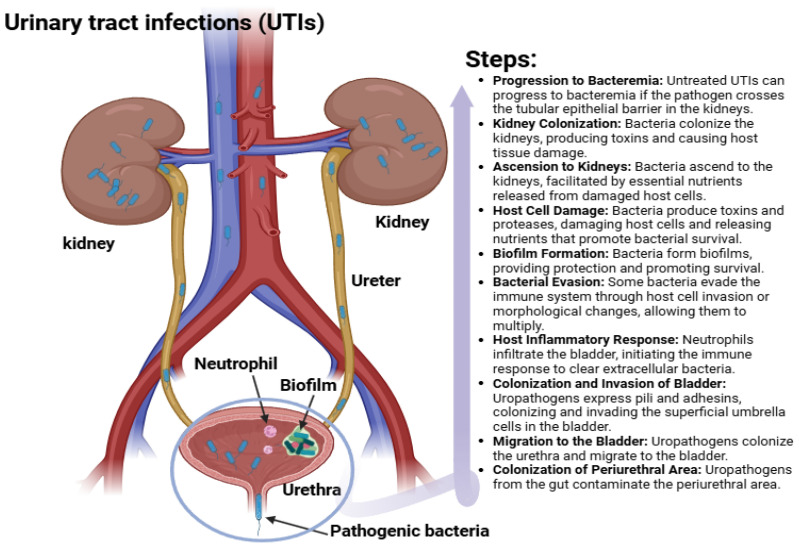
UTIs in women [28,29,30,31,32,33,34,35,36,37].

UTIs in females present a comprehensive progression through the urinary system, including the kidneys, ureters, bladder, and urethra (Figure 1). The image highlights key anatomical structures and the involvement of pathogenic bacteria, biofilm formation, and neutrophil response in the bladder. The progression of UTIs follows several steps, starting with the migration of bacteria from the periurethral area, often originating from the gut, into the urethra and bladder [18]. Once in the bladder, bacteria attach and invade bladder cells via pili and adhesins, evade immune defenses through cell invasion or morphological changes, and form biofilms that provide protection and enhance survival [1,2,3,4,5]. If left untreated, bacteria can ascend to the kidneys, facilitated by nutrients from damaged host cells, leading to colonization, toxin production, and tissue damage. In severe cases, bacteria can cross the tubular epithelial barrier in the kidneys, resulting in bacteremia. The body’s immune response, particularly through neutrophil infiltration, attempts to combat the infection by clearing extracellular bacteria [25].

UTIs in females are primarily attributed to the bacterium *E. coli*, which typically emanates from the gastrointestinal tract. Upon entering the urethra, *E. coli* can ascend to the bladder, resulting in cystitis, an inflammatory condition of the bladder marked by manifestations such as increased frequency of urination, dysuria, and pain in the lower abdominal region [22]. UTIs rank among the most prevalent bacterial infections, with approximately fifty percent of women experiencing at least one UTI during their lifetime [23]. The anatomical configuration of females, including a comparatively shorter urethra and the close proximity of the urethral opening to the anus, enhances their vulnerability to such infections [24,25]. Furthermore, factors such as sexual intercourse and certain contraceptive approaches can further heighten the likelihood of developing UTIs [26]. The timely recognition of symptoms is vital for swift intervention, which can avert complications and enhance clinical outcomes [27]. Comprehending the underlying mechanisms of UTIs and their associated risk factors is imperative for the formulation of effective prevention and management strategies for women [38,39].

UTIs are broadly categorized into three types: uncomplicated UTIs (uUTIs), complicated UTIs (cUTIs), and asymptomatic bacteriuria, each based on patient characteristics and clinical presentations (Table 1) [38]. Uncomplicated UTIs typically occur in women without anatomical or functional urinary tract abnormalities, presenting with classic symptoms like dysuria and frequent urination [23]. In contrast, cUTIs arise in individuals with predisposing factors such as pregnancy, diabetes, or kidney disease, and may involve more severe infections like pyelonephritis, while asymptomatic bacteriuria is the presence of significant bacteria in the urine without clinical symptoms, often identified during routine testing [40].

In instances of asymptomatic bacteriuria, characterized by the presence of bacteria in the urine without any clinical manifestations, laboratory verification is essential for precise diagnosis and management [28]. The dependence on urinalysis is emphasized by its function in assessing the physical, chemical, and microscopic characteristics of urine, which aids in detecting the occurrence of infection [29]. Quantitative urine cultures hold particular significance, as they offer the most precise evaluation of bacteriuria, particularly in individuals who are asymptomatic [30]. The lack of symptoms necessitates this laboratory-oriented methodology, since inappropriate treatment of asymptomatic bacteriuria can result in superfluous healthcare expenditures and safety concerns [31]. Consequently, urinalysis outcomes are pivotal for the diagnosis of asymptomatic UTIs, directing healthcare professionals in their clinical judgments when there are no symptoms present [32].

The meticulous collection of urine specimens is of paramount importance, especially for female patients, owing to the anatomical closeness of the external urethral orifice to the vagina, which heightens susceptibility to contamination from vaginal fluids. Such contamination can profoundly influence the validity of urinalysis outcomes, potentially resulting in erroneous diagnoses [33]. To alleviate these concerns, the midstream urine collection technique is advocated, as it entails discarding the initial flow of urine to diminish the chances of contamination [34]. Moreover, the establishment of explicit guidelines for urine sample collection is crucial, as these protocols should incorporate hygiene standards and appropriate collection methodologies specifically designed for female patients [35]. Furthermore, the education of patients is integral in ensuring that they comprehend the proper procedures for urine specimen collection, which can improve the integrity of urinalysis findings [36]. Despite the implementation of preparatory vulval cleansing, research has indicated no substantial variance in contamination levels between specimens obtained with or without this procedure [37]. Consequently, prioritizing midstream collection along with robust patient education may represent more efficacious approaches for acquiring precise urine samples in females.

The higher incidence of post-coital UTIs in females is often attributed to the shorter anogenital distance, which is considered a primary anatomical determinant of UTI susceptibility [17]. The shorter distance between the anus and urethra in females facilitates the easier migration of uropathogens, primarily *E. coli*, from the perineal and rectal regions to the urethral opening during sexual activity [16]. This anatomical predisposition, combined with mechanical factors such as friction and urethral irritation during intercourse, increases the likelihood of bacterial colonization and subsequent infection of the bladder. Additionally, the shorter urethra in females provides a more direct pathway for bacteria to ascend into the bladder, leading to a higher risk of infection compared to males [25].

The innate female attribute of urothelial shedding in response to stress has been suggested as a potential primary trigger for UTIs. The urothelium, which serves as a critical barrier against uropathogens, undergoes a dynamic process of shedding and regeneration, particularly under physiological and pathological stress conditions. According to recent studies, the stress-induced shedding of urothelial cells may expose immature basal and intermediate cells, which lack the fully developed protective features of mature umbrella cells [41]. These immature cells may exhibit weaker intercellular junctions and reduced expression of key antimicrobial peptides, making them more susceptible to bacterial adhesion and invasion. Consequently, the compromised integrity of the urothelial barrier due to stress-induced shedding may facilitate bacterial entry, colonization, and the establishment of infection, thereby increasing the risk of UTIs in females [25].

Moreover, the role of urothelial immaturity in UTI susceptibility is further supported by the observation that mature umbrella cells possess specialized features, such as a highly glycosylated apical membrane, which provide an effective defense against bacterial adherence [13]. In contrast, the immature urothelium, exposed following stress-induced shedding, may lack these protective adaptations, rendering it more vulnerable to uropathogenic *E. coli* and other bacteria. Additionally, stress-related hormonal changes, such as elevated cortisol levels, can impair urothelial regeneration and immune responses, further exacerbating susceptibility to infections. Understanding the interplay between urothelial shedding, maturation, and host defense mechanisms could provide valuable insights into developing targeted preventive and therapeutic strategies to mitigate UTI risk in females [25].

The leukocyte count is the primary parameter utilized in the diagnosis of UTIs. Typically, a leukocyte level exceeding 10 leukocytes/mm^3^ signifies the presence of an infection. Nevertheless, in pregnant patients, the threshold is elevated to >20 leukocytes/mm^3^, attributable to the physiological alterations accompanying pregnancy. Sample contamination from vaginal secretions, mucus, or lactic acid bacteria may obfuscate the analytical results. For instance, such contamination could result in the erroneous identification of mucus strands or excessive bacterial proliferation within urine sediment. This may occasionally lead to diagnostic errors, such as the misinterpretation of mucus as proteinuria [42]. Menstrual flow, postpartum discharge, or other forms of uterine hemorrhage can also compromise the integrity of the sample by introducing erythrocytes into the urine, thereby complicating result interpretation [43,44,45,46,47]. In these scenarios, it is imperative to obtain a thorough patient history and perform a meticulous reanalysis to ensure diagnostic precision. Urinalysis alone is inadequate for diagnosing a UTI in the absence of clinical symptoms. A urine culture is highly advocated to either confirm or exclude infection. For optimal results, the culture specimen should be obtained in a sterile container, ideally from the first morning urine, to minimize the risk of contamination. A bacterial concentration of ≥105 CFU/mL substantiates the diagnosis of an infection. An antibiogram is conducted to verify the efficacy of particular antibiotic regimens, thereby ensuring that the prescribed antibiotics are effective against the identified pathogenic strain [48].

**Table 1:** Categories of UTI [18,25,30,46,47,48].

Aspect	uUTI	cUTI	Asymptomatic Bacteriuria
Patient popultion	Women with structural or functional issues in the urinary tract	Patients with pregnancy, poorly controlled diabetes, hospital-acquired infections, kidney disease, prior kidney transplant, kidney stones, anatomical or functional abnormalities (e.g., genital prolapse), use of immunosuppressants, indwelling catheter, or postoperative complications	Any individual, whether pregnant or non-pregnant
Clinical symptoms	Classic UTI symptoms like painful urination (dysuria), frequent urination (pollakiuria), and lower abdominal pain (suprapubic pain); pyelonephritis may occur with additional flank or groin pain and fever	Recurring UTI symptoms in individuals with risk factors (as listed above), pyelonephritis, or history of treatment with NSAIDs * and/or antibiotics	No symptoms
Diagnostic approach	No testing needed for first UTI episode; for pyelonephritis or recurrent UTI, urinalysis and culture are recommended; for suspected pyelonephritis, ultrasound is advised	Urinalysis and urine culture required; for suspected pyelonephritis, kidney ultrasound and assessment of postvoid residual urine; in pregnancy, cervical length should be measured; for hospitalized patients with fever >38.3 °C, blood cultures are recommended (2×)	Diagnostics are unnecessary unless a urological procedure is planned that risks damage to the bladder mucosa
Microbial cause	Mainly *E. coli* (over 80%), but also *Proteus mirabilis*, *Klebsiella* species, *Staphylococcus saprophyticus*, and *Enterococcus faecalis*	*E. coli* (including extended-spectrum beta-lactamase (ESBL) strains), *Enterococcus faecalis*, other *Enterobacterales*, and *Pseudomonas aeruginosa*	Any bacterial species, with ≥10^5^ bacteria per milliliter of urine

* NSAIDs = nonsteroidal anti-inflammatory drug.

UTIs are among the most common infectious diseases affecting individuals across all age groups, with a particularly high prevalence in the elderly population [49]. The growing incidence and recurrence of UTIs, combined with the global rise in antibiotic-resistant microorganisms, pose substantial challenges for the accurate diagnosis and effective management of both lower and upper UTIs in clinical practice. Complicating matters further, the presence of atypical or asymptomatic clinical presentations highlights the critical need for early detection to mitigate the risk of septicemia and long-term health complications [50].

Current diagnostic practices for UTIs primarily rely on clinical symptom assessment, nitrite strip tests that indicate the presence of urinary pathogens, and the measurement of leukocyte counts in urine samples. However, urine culture remains the gold standard for diagnosis, despite being time-consuming and costly [51]. To overcome these limitations, ongoing efforts have focused on identifying novel biomarkers to enhance the accuracy and speed of UTI diagnostics. Potential biomarkers under investigation include leukocyte esterase, heparin-binding protein, C-reactive protein (CRP), procalcitonin, lactoferrin, interleukins, elastase alpha (1)-proteinase inhibitor, secretory immunoglobulin A, α-1 microglobulin (α1Mg), xanthine oxidase, soluble-triggering receptor expressed on myeloid cells-1, myeloperoxidase, and tetrazolium nitroblue test (TNB) products [52]. However, the low concentration of these biomarkers in biological samples presents a significant challenge for their quantitative assessment.

The accurate identification of the causative agents of infection is crucial for improving patient outcomes, guiding appropriate therapeutic interventions, and implementing effective infection prevention strategies. Numerous diagnostic approaches have been proposed to address the limitations of the current methods. Given the unique capabilities of nanotechnology, its application in UTI diagnostics offers a promising pathway to overcome the existing challenges, enabling faster, more precise, and cost-effective detection methods [53].

## 4. Characteristic Patient Groups

UTIs are particularly prevalent among peri- and postmenopausal women, and the primary reasons for this increased susceptibility include hormonal changes, particularly estrogen insufficiency, and the aging of connective tissues, which can lead to urinary incontinence and pelvic organ prolapse [54]. During the perimenopausal period, several factors further contribute to the risk of UTIs. These include urinary incontinence, which can impede proper hygiene, atrophy of vaginal mucous membranes, which increases the likelihood of vaginal infections that may spread to the urinary tract, and anterior vaginal prolapse, which can prevent complete bladder emptying. Additionally, the prevalence of asymptomatic bacteriuria significantly rises during this stage of life, affecting 4–19% of peri- and postmenopausal women, compared to only 1.5% in premenopausal women [55].

One of the key contributors to both urinary incontinence and UTIs in peri- and postmenopausal women is estrogen deficiency. The topical vaginal application of estrogens, as opposed to systemic administration, has been shown to significantly reduce the risk of bacteriuria, with an odds ratio (OR) of 0.3 (95% confidence interval [CI]: 0.13–0.68). Consequently, guidelines published by various research associations recommend the use of topical estrogens in peri- and postmenopausal women to prevent UTIs [56].

Diabetes is another significant risk factor for UTIs in postmenopausal women. Research involving a total of 256,725 females with type 2 diabetes revealed a marked increase in UTI diagnoses starting from the ages of 45–49 years, with a 100% increase in prevalence for women within this age range and an additional 80% rise for those aged 50–54 years [57]. A separate study compared two groups of women aged 55–75 who had been diagnosed with acute UTIs: 901 diabetic patients and 913 controls. The results demonstrated that diabetes caused a twofold increase in UTI risk in postmenopausal women (OR = 2.2; 95% CI: 1.5–3.1). Significant risk factors included the use of oral pharmacotherapy or insulin treatment (OR = 2.8 and 2.7, respectively), and the presence of type 2 diabetes (OR = 2.2). Interestingly, disease duration and glycemic control, as assessed by glycated hemoglobin (HbA1c) levels, were not significant risk factors. Furthermore, women aged 57 and older undergoing surgical treatments were at a higher risk of UTIs [58].

Diabetes mellitus, particularly when uncontrolled, significantly increases the risk of infections in both the urinary and reproductive tracts. This includes infections of the vulva, vulvar vestibule, and vagina. Studies have revealed that 14% of women with type 1 diabetes and 23% of those with type 2 diabetes are diagnosed with UTIs. The most critical risk factors in this population are poor glycemic control and glycosuria. Moreover, women with a longer disease duration, particularly those in the perimenopausal phase, are at a higher risk of infection [59]. One notable study of 1357 women with type 1 diabetes found a higher prevalence of acute cystitis, acute vaginitis, and acute vulvitis. In another study involving 241 women with type 1 diabetes, the primary risk factors for symptomatic infections included sexual activity, the use of oral contraceptives, and the presence of microangiopathy. Additionally, urinary incontinence, which occurs more frequently in women with diabetes than in the general population, was identified as a significant contributing factor [60].

Asymptomatic bacteriuria is also more prevalent in women with type 2 diabetes compared to healthy controls. Studies indicate that 20% of diabetic patients with asymptomatic bacteriuria progress to having a symptomatic UTI within six months. A study involving 348 women with type 2 diabetes identified asymptomatic bacteriuria as a primary risk factor for the development of symptomatic infections, which in turn may lead to a decline in renal function. Given these findings, routine urine culture screening may be beneficial for diabetic patients, particularly those with type 2 diabetes [61].

Epileptic women are another population at heightened risk of UTIs, with studies reporting a UTI prevalence of approximately 58% compared to 42% in the general population (*p* < 0.0001). The increased UTI susceptibility in this group has been attributed to the use of antiepileptic drugs (AEDs), which exhibit immunomodulatory effects. Notably, women receiving treatment with phenytoin (OR = 1.78; 95% CI: 1.24–2.55; *p* = 0.001), primidone (OR = 1.73; 95% CI: 1.21–2.49; *p* = 0.002), carbamazepine (OR = 1.61; 95% CI: 1.33–1.96; *p* < 0.0001), and valproate (OR = 1.52; 95% CI: 1.28–1.82; *p* < 0.0001) experience higher rates of UTIs. These drugs may impair immune function, thereby increasing susceptibility to urinary tract infections. Such findings emphasize the need for clinicians to consider UTI prevention strategies when prescribing AEDs to epileptic women [62].

Patients who use indwelling urinary catheters or require intermittent self-catheterization are also at increased risk of UTIs. In this population, the frequency of UTIs ranges from 15.4% to 86.6% annually. Catheter use compromises the integrity of the urinary tract’s natural defenses, promoting bacterial colonization. Preventive strategies, including the application of antiseptic products, have been shown to reduce UTI incidence in these patients [63].

The perioperative period introduces another set of risk factors for UTIs. Post-surgical patients, particularly women undergoing urological, gynecological, and abdominal procedures, face an elevated UTI risk. Factors contributing to this increased risk include advanced age (above 57–60 years), diabetes, obesity, immunosuppressant therapy, and the need for blood transfusions due to surgical complications. Studies highlight the benefits of administering prophylactic antibiotics to patients with asymptomatic bacteriuria who require catheterization for surgery. This approach reduces the risk of developing a symptomatic infection (relative risk [RR] = 0.20; 95% CI: 0.13–0.31). Women undergoing gynecological surgery, including cesarean sections, are often catheterized for several hours to several days post surgery. In cesarean sections, catheters are generally removed after a few hours of anesthesia, whereas for pelvic organ prolapse repair, catheterization may last 2–3 days. This prolonged catheterization period increases the risk of UTIs, underscoring the importance of preventive measures during the perioperative period [64].

Pregnancy introduces a series of factors conducive to UTIs. Pregnant women tend to have more basic urine, experience urine flow obstructions (especially in the later stages of pregnancy), and are more likely to develop proteinuria, diabetes, and anemia. Obtaining a clean urinalysis sample becomes more challenging as pregnancy progresses, particularly in the third trimester, leading to the detection of proteins and bacteria in samples that do not always indicate infection [65]. Often, these findings result from contamination by vaginal secretions. Protein in the sample may be caused by mucus contamination, but in hypertensive patients, it suggests proteinuria characteristic of preeclampsia. When multiple bacteria are observed in the urine sediment without an accompanying elevation in leukocyte levels, lactic acid bacteria are typically responsible, making leukocyte levels a key factor in differentiating between UTIs and other conditions in pregnant women [66].

Asymptomatic UTIs affect 2–8% of pregnant women, though some studies suggest that up to 50–60% of pregnant women may be diagnosed with UTIs. Research also indicates an association between asymptomatic bacteriuria and an increased risk of preterm birth [67]. UTIs are diagnosed more frequently in women with gestational hypertension, and they are linked to a higher risk of intrauterine growth restriction, premature birth, and cesarean sections [68]. However, it is important to note that a UTI diagnosis alone does not necessitate specific obstetric interventions [69]. Recurrent UTIs affect 25% of pregnant women diagnosed with a UTI, and 4–5% of these cases progress to pyelonephritis [70]. Additionally, a maternal UTI is a significant risk factor for the child’s development of UTIs, with a 30% occurrence rate compared to 6.8% in the general population (OR = 5.9; 95% CI: 1.9–18.3; *p* = 0.001) [71].

Pregnancy-associated immunological modifications are instrumental in inhibiting the progression of asymptomatic bacteriuria to symptomatic UTIs in individuals who are pregnant [65]. Throughout the gestational period, the maternal immune system experiences profound alterations to facilitate fetal growth, while concurrently upholding a robust defense against infectious agents [67]. These modifications encompass a transition towards an anti-inflammatory milieu during the initial stages of pregnancy to encourage fetal tolerance, succeeded by a pro-inflammatory environment in the later phases to promote the onset of labor. Notably, pregnancy is characterized by variations in the levels of immune cells such as regulatory T cells, which are pivotal in sustaining immune tolerance, alongside an augmented synthesis of antimicrobial peptides that establish a protective barrier against uropathogens [72]. Furthermore, hormonal fluctuations, particularly the elevation of progesterone and estrogen, significantly impact immune responses by regulating cytokine production and enhancing the antimicrobial characteristics of the urinary tract [73]. It is posited that these immunomodulatory phenomena contribute to the relatively low incidence of progression from asymptomatic bacteriuria to symptomatic UTIs, which is observed in merely 1–2% of pregnancies, despite asymptomatic bacteriuria being present in up to 13% of individuals who are pregnant [74].

Pregnancy induces profound alterations in the urinary tract environment, including a substantial increase in urinary proteoglycans and glycocalyx, which play a crucial role in bacterial entrapment and immune defense. A notable change is the 30-fold rise in urinary proteoglycan content, which is functionally analogous to neutrophil extracellular traps (NETs), effectively capturing and preventing bacterial colonization [75,76]. This mechanism suggests that the lower incidence of symptomatic UTIs in pregnant women is not merely due to systemic immune modulation, but also to localized, biochemical changes in urine composition that create an unfavorable environment for bacterial proliferation.

Additionally, pregnancy is characterized by a significant increase in circulating syndecan-1, a crucial component of the glycocalyx, which serves as a protective barrier in endothelial tissues [75]. Plasma syndecan-1 levels are markedly elevated in normal pregnancy compared to non-pregnant states, with a further gestational age-dependent increase. In uncomplicated pregnancies, syndecan-1 concentrations rise from 1280 ng/mL (preterm) to 1786 ng/mL (term), indicating its role in maintaining endothelial integrity. Elevated levels of syndecan-1 in pregnancy correlate with enhanced glycocalyx function, which extends to the urinary tract, reinforcing the endogenous barrier against bacterial adhesion and preventing the progression of asymptomatic bacteriuria to clinically significant UTIs.

Furthermore, urinary levels of bikunin, lactoferrin, and syndecan-1 are significantly increased during pregnancy [75]. Bikunin, a proteoglycan with broad-spectrum antimicrobial and anti-inflammatory properties, is a well-established inhibitor of bacterial adherence to urothelial surfaces. Lactoferrin, a potent iron-chelating glycoprotein, limits bacterial growth by depriving pathogens of essential nutrients, while syndecan-1 contributes to the stabilization of the urothelial glycocalyx, reducing bacterial penetration and subsequent infection risk. These findings underscore a pregnancy-adapted biochemical defense mechanism that significantly reduces UTI susceptibility.

Interestingly, the exogenous instillation of proteoglycans has been adopted as a therapeutic strategy to prevent UTIs in neurogenic and hospitalized patients, highlighting the translational significance of these endogenous protective factors [76]. The presence of these naturally occurring proteoglycans in pregnancy raises the possibility of novel interventions for high-risk populations, including pregnant individuals with preexisting urinary tract abnormalities or recurrent UTI history.

Beyond the urinary tract, pregnancy also induces a dramatic increase in neutrophil counts, which has not been previously discussed in our manuscript. Neutrophils, through NETosis, contribute to an additional layer of antimicrobial defense. Given that pregnancy is associated with an elevated neutrophil count and heightened immune readiness, it is plausible that these immunological shifts further enhance resistance against symptomatic UTIs [75,76].

Based on this, we propose several testable hypotheses to advance the field that could provide deeper insights into the protective mechanisms against UTIs in pregnant women. One key area of investigation is whether urinary syndecan-1 levels correlate inversely with symptomatic UTI risk, which could establish its potential role as a biomarker for predicting susceptibility to infection. Additionally, exploring the role of glycosaminoglycan-rich urinary microenvironments in bacterial entrapment and clearance may reveal novel antimicrobial defense mechanisms inherent to pregnancy. Furthermore, assessing differences in urinary proteoglycan composition between pregnant individuals with and without recurrent UTIs could provide valuable insights into potential diagnostic markers and therapeutic strategies for preventing infections in high-risk populations.

The escalating incidence of UTIs among diverse demographic cohorts, notably peri- and postmenopausal women, individuals with diabetes mellitus, patients with epilepsy, and pregnant women, highlights the imperative for customized preventive and therapeutic interventions (Table 2) [77,78,79,80]. Overall, the application of topical estrogen has demonstrated efficacy in diminishing the risk of UTIs in menopausal women, whereas maintaining appropriate glycemic control is critically important for individuals with diabetes [81]. Healthcare practitioners should exercise vigilance in the management of high-risk populations, such as those necessitating catheterization or undergoing treatment with antiepileptic drugs, to reduce the likelihood of recurrent infections [82]. Additional scholarly inquiry is essential to identify novel strategies for the prevention and management of UTIs, particularly within groups exhibiting distinctive risk characteristics [83].

## 5. Advancements in UTI Treatment

### 5.1. Antibiotic Treatment

The diagnosis of UTIs is commonly established when bacterial concentrations in urine reach ≥ 10^5^ colony-forming units per milliliter (CFU/mL), as this threshold indicates a significant bacterial presence that typically warrants antibiotic treatment [44]. However, bacterial concentrations below 10^3^ CFU/mL are generally considered to fall within the range that the innate immune system can effectively manage without pharmacological intervention. Innate immune defenses, including antimicrobial peptides, neutrophil infiltration, and urothelial shedding, play a crucial role in clearing low-level bacterial colonization, preventing progression to symptomatic infection [46]. The overuse of antibiotics in such low bacterial concentrations may contribute to antimicrobial resistance and disrupt the natural microbiota of the urinary tract. Therefore, careful clinical evaluation and consideration of patient symptoms are essential in determining whether antibiotic treatment is necessary, emphasizing the importance of balancing antimicrobial stewardship with effective infection control strategies [65].

The fundamental principle in the management of bacterial infections, including UTIs, is the implementation of effective antimicrobial therapy, wherein the judicious application of antibiotics is paramount, particularly in light of the elevated incidence of UTIs among the female population [35,69]. Healthcare professionals bear a significant responsibility in guaranteeing that patients receive appropriate treatment while complying with established antibiotic stewardship protocols and clinical practice guidelines [65,70]. The selection of specific antibiotics and the duration of therapeutic intervention are contingent upon various factors, including the anatomical site of infection, the severity of the clinical presentation, and considerations pertaining to both the bacterial pathogen and host’s characteristics. For instance, in the context of prostatitis and epididymo-orchitis, it is imperative to attain adequate tissue concentrations of the administered antibiotics [55,71].

For uncomplicated UTIs, the Infectious Diseases Society of America (IDSA) endorses the use of nitrofurantoin, trimethoprim-sulfamethoxazole (TMP-SMX), or fosfomycin for a treatment duration of 3 to 5 days (Table 3) [72]. Nitrofurantoin, which exhibits efficacy against *E. coli*, demonstrates diminished potency against other Gram-negative organisms such as *Klebsiella* and *Pseudomonas*. Clinicians are advised to remain vigilant regarding the infrequent yet severe adverse effects, including pulmonary fibrosis and interstitial pneumonitis, which may particularly affect susceptible patient populations, such as those with chronic obstructive pulmonary disease (COPD). TMP-SMX is recognized for its high efficacy in the eradication of UTIs; however, its application is advised only when local resistance rates are maintained below 20%. This agent is contraindicated in individuals with documented sulfa allergies. Although fosfomycin presents a feasible alternative for uncomplicated UTIs, it may exhibit marginally reduced effectiveness relative to other available antimicrobials [56,73].

Quinolones, which have historically been utilized in the treatment of UTIs, are now frequently eschewed in uncomplicated scenarios due to the increasing prevalence of resistance and the necessity to reserve these agents for more complicated infections. In instances where first-line therapeutic strategies prove ineffective, the predominant underlying factor is often bacterial resistance, thereby necessitating a subsequent urine culture and the exploration of alternative antibiotic options, such as quinolones or cephalosporins (Table 3) [74]. The rise in extended-spectrum β-lactamase (ESBL)-producing bacteria in the context of community-acquired UTIs, observed in up to 7% of affected cases, underscores the need for individualized antibiotic therapy predicated on culture findings, which may involve the utilization of carbapenems in instances of heightened resistance. Ensuring adequate hydration and addressing any urinary obstructions or foreign bodies are critical adjunctive measures in the overall treatment strategy [77].

### 5.2. Non-Antibiotic Prophylactic Treatment—Immunomodulation

The predominant non-antibiotic management strategies for recurrent UTIs that have been extensively researched encompass cranberries, probiotics, d-mannose, methenamine hippurate, estrogens, intravesical glycosaminoglycans, immunostimulants, OM-89, and Chinese herbal medicine (CHM). Notably, CHM has garnered significant scholarly interest for its potential therapeutic and preventive effects concerning recurrent UTIs.

*Chinese herbal medicine*. Chinese herbal constituents, such as Huang Lian (*Coptis chinensis* Franch), have exhibited inhibitory effects against uropathogenic microorganisms, particularly *E. coli*, while also showcasing anti-inflammatory characteristics. Additionally, another herbal formulation, Compound Salvia Plebeia Granules (CSPG), have been documented to diminish the adhesion of *E. coli* to the urothelial cells of the bladder. In experimental studies involving male rats, the intragastric administration of CSPG at dosages ranging from 20 to 40 g/kg produced diuretic, antipyretic, and analgesic responses, along with an observed inhibition of *E. coli* proliferation, with a minimal inhibitory concentration (MIC) established at 0.25 g/mL. Subsequent in vitro investigations into the principal berberine alkaloids (berberine, coptisine, and palmatine) derived from *C. chinensis* Franch indicated a reduction in the growth rate of *E. coli* [86].

Several targeted CHM formulations, including Er Xian Tang, Bai Tou Weng Tang, and San Jin Wan, have undergone evaluation in female populations suffering from recurrent UTIs. A systematic review conducted by Cochrane, encompassing seven randomized controlled trials (RCTs) with a total of 542 participants, scrutinized the efficacy of CHM in the treatment and prevention of recurrent UTIs. Among these studies, three directly compared CHM to antibiotic interventions, whereas two explored the synergistic effects of combining CHM with antibiotics against antibiotics administered in isolation. The results indicate that CHM alone yielded a statistically significant reduction in UTI recurrence (relative risk (RR) 0.28, 95% CI 0.09–0.82), while the combination of CHM and antibiotics was more effective in preventing recurrences compared to antibiotics alone (RR 0.53, 95% CI 0.35–0.80) [82].

While the preliminary data are encouraging, these findings underscore the imperative for additional high-quality RCTs to validate the efficacy and safety of CHM in the context of UTI management.

*Cranberry*. The empirical evidence regarding the efficacy of cranberries and cranberry-derived products for the prophylactic management of urinary tract infections (UTIs) remains inconclusive, and scant evidence supports its therapeutic role in the management of acute UTIs. Cranberry, scientifically classified within the family Ericaceae, is recognized by its botanical names *Vaccinium macrocarpon*, *Vaccinium oxycoccos*, and *Vaccinium erythrocarpum*. The composition of the berries is predominantly aqueous (88%), accompanied by a complex array of organic acids, fructose, ascorbic acid, flavonoids, anthocyanidins, proanthocyanidins, catechins, and triterpenoids. Anthocyanidins and proanthocyanidins are classified as tannins (polyphenols), which serve as a natural defense mechanism in plants against microbial infections and are considered the most clinically significant components in mitigating UTIs among women. Historically utilized since the 17th century for a variety of therapeutic applications, including gastrointestinal and hepatic ailments, scurvy, and neoplasms, cranberry has been a widely adopted intervention for UTIs for nearly a century, particularly prior to the introduction of antibiotic therapies [84,85].

*Nonsteroidal anti-inflammatories*. The initial interactions between the host and the pathogen, as well as the intensity of the host’s acute inflammatory response during the first 24 h after infection, are likely to influence the likelihood of experiencing chronic cystitis (characterized by persistent symptoms in contrast to the complete alleviation of symptoms typically associated with acute urinary tract infections), as evidenced by findings in murine models [55]. The occurrence of pronounced pyuria, damage to the bladder mucosa, and heightened levels of specific cytokines (IL-5, IL-6, IL-8, and G-CSF) emerge as significant predictors for the onset of chronic cystitis. Notably, the pre-administration of dexamethasone, a corticosteroid used to suppress inflammation, prior to the induction of a urinary tract infection significantly reduced the incidence of chronic cystitis in 103 murine subjects (2 of 17 vs. 13 of 18; *p* < 0.001). The chronic inflammatory response in the bladder resulting from prolonged exposure to bacterial agents leads to lymphonodular hyperplasia and the infiltration of B cells within the bladder submucosa, and has been observed in studies involving patients with neurogenic bladder and recurrent urinary tract infections [86]. Elevated levels of serum cytokines and growth factors pertinent to the development of monocytes and macrophages were reported in women with recurrent urinary tract infections in comparison to those without. Moreover, the expression of cyclooxygenase 2 (Cox2) mRNA was found to increase in murine models of urinary tract infection by as much as 50-fold within 24 h post *E. coli* infection, and COX2 expression was also elevated in urinary particulates obtained from patients suffering from urinary tract infections. The inhibition of COX2 in a murine model resulted in a marked reduction in the severity of pyuria following urinary tract infection, alongside diminished bacterial loads in the bladder and a lower incidence of recurrent chronic cystitis (31% (8 of 26) versus 77% (20 of 26); *p* < 0.05). These findings imply the potentially advantageous role of nonsteroidal anti-inflammatory drugs or COX2 inhibitors in the management of acute urinary tract infections and in the prevention of the recurrence of urinary tract infections [86].

*OM-89*. OM-89 represents an immunomodulatory pharmaceutical agent. This therapeutic agent demonstrates efficacy against *E. coli* infections, which account for 70–80% of all urinary tract infections [87]. In a cohort of women experiencing recurrent urinary tract infections, those administered OM-89 for a duration of six months exhibited a twofold decrease in subsequent recurrence rates (67.3% versus 32.7%). Nevertheless, uncontrolled diabetes markedly diminished its therapeutic effectiveness [88]. In a multicenter, double-blind clinical trial encompassing 453 women, a 34% reduction in urinary tract infections was documented following three months of initial treatment, along with a 10-day booster regimen of OM-89. A similar treatment paradigm was employed in a retrospective analysis involving 79 patients, wherein *E. coli* was identified as the predominant pathogen in 49% of the study population. Among those infected with *E. coli*, 63% exhibited a positive treatment response, while 53% of the overall population also demonstrated a favorable outcome [89].

The efficacy of OM-89 was further substantiated in a study focused on menopausal women. A clinical trial was conducted involving a cohort of patients with an average age of 66 years. The incidence of recurrent infections within this group decreased from 3.4 to 1.8 (a reduction of 65%) following the administration of immunomodulatory treatment [90].

The oral immunomodulatory regimen of OM-89 for the prophylaxis of recurrent urinary tract infections is endorsed by the EAU for uncomplicated urinary tract infections in women (strong evidence, highest recommendation level, 1a). This treatment is effective in mitigating the frequency of recurrent infections, alleviating patient symptoms, reducing antibiotic prescriptions, and minimizing the risk of developing antibiotic resistance. For the prevention of recurrent urinary tract infections, OM-89 is administered daily prior to meals, over a course of 90 days. The drug can be concomitantly utilized alongside antibiotic therapy during the acute phase of an infection, without necessitating prior urine culture results, given its capacity to elicit a robust immune response, not only to *E. coli* but also to other pathogens implicated in urinary tract infections. OM-89 is distinguished by the highest level of evidence among all non-antibiotic strategies for the prevention of urinary tract infections [91].

*Estrogen hormones.* Urinary tract infections (UTIs) occur due to an imbalance between the body’s natural defense mechanisms and the virulence of bacteria. Estrogen hormones, particularly 17β-estradiol (E2), play a critical role in regulating cell growth and differentiation. While estrone is also produced, it primarily comes from the conversion of androstenedione in peripheral tissues. The hydroxylation of these hormones results in the formation of estriol, which is the main form of estrogen present in urine. Estrogen receptors, specifically ERα and ERβ, mediate epithelial differentiation and maintenance. ERα is predominantly found in the vagina, while ERβ is more prevalent in the bladder. These receptors are also found in the pubococcygeus and pelvic floor muscles. After menopause, declining estrogen levels cause chemical and structural changes, such as reduced urinary flow, increased residual urine volume, and an elevated vaginal pH. This, combined with the loss of commensal lactobacilli in the vagina, increases susceptibility to UTIs. Although estrogen plays a crucial role in enhancing the natural defenses of the lower urinary tract against UTIs, the precise mechanisms remain unclear [92].

In premenopausal women, circulating estrogens promote vaginal colonization by lactobacilli, which is essential for maintaining vaginal health. Estrogen increases the proportion of glycogen-producing vaginal epithelial cells, which serve as a substrate for lactobacilli. Lactobacilli convert glycogen into lactic acid, hydrogen peroxide, and antimicrobial peptides like bacteriocin, maintaining an acidic vaginal pH (below 4.5), which inhibits the growth of uropathogens. After menopause, the decline in estrogen levels raises the vaginal pH above 4.5, reducing lactobacilli and allowing gut bacteria, such as *E. coli*, to colonize the vagina more easily [93].

A randomized controlled trial involving 93 postmenopausal women found that intravaginal estriol cream significantly reduced UTI incidence compared to a placebo (0.5 vs. 5.9 episodes per patient per year). Lactobacilli returned in 61% of estriol users compared to none in the placebo group, and vaginal pH dropped from 5.5 to 3.8 in the estriol group [94]. Vaginal colonization by *Enterobacteriaceae* also decreased. However, 20% of estriol users experienced adverse effects, including vaginal irritation and itching, leading to treatment discontinuation. Patients should be informed about these potential side effects to improve treatment adherence [95].

In the bladder and urethra, estrogen regulates cell growth and differentiation and may influence the production of antimicrobial peptides. Studies show that bacterial contact with urothelial cells triggers the release of cathelicidin, an antimicrobial peptide, into the urine [96]. Postmenopausal women have significantly lower serum cathelicidin levels, which correlate positively with estradiol levels. After two weeks of estradiol supplementation in postmenopausal women, the expression of several antimicrobial peptides increased in urinary cells. Additionally, increased estrogen levels improve urethral closure pressure, enhancing continence and reducing postvoid residual volume, factors associated with a lower risk of recurrent UTIs [97].

Oral estrogen therapy is commonly prescribed to young postmenopausal women (ages 50–60) to alleviate menopausal symptoms like hot flashes, night sweats, vaginal dryness, and insomnia. Alongside these benefits, it may also help to prevent conditions such as osteoporosis, ischemic heart disease, and UTIs, which pose a higher risk in postmenopausal women. However, studies indicate that oral estrogen therapy does not significantly reduce the frequency of recurrent UTIs (RR 1.08, 95% CI 0.88–1.33) or increase lactobacilli levels in the urinary tract [98].

A meta-analysis of two studies revealed a reduction in vaginal pH (from an average of 6.5 to 5.5) after oral estrogen use, but this came with increased side effects, such as breast tenderness and mild vaginal bleeding. Due to these findings, local estrogen preparations, rather than systemic oral therapy, are recommended for preventing recurrent UTIs. It is important to note that oral estrogen therapy has absolute contraindications, including a history of breast or endometrial cancer, thromboembolic disorders, and acute liver disease, as it can worsen cholestasis [99].

Four randomized controlled trials involving 2798 postmenopausal women examined oral estrogen (with or without progestogens) versus a placebo for UTI prevention, but no significant benefit in reducing UTI recurrence was observed. Local estrogen application is preferred for this purpose [100].

*Other methods*. According to a 2017 Cochrane database analysis, further research is needed to fully understand the effectiveness of probiotics in reducing urinary tract infections (UTIs), particularly in patients with bladder function disorders. Managing chronic UTIs and preventing recurrences remains a significant challenge. D-mannose has shown potential in preventing recurrent UTIs by inhibiting bacterial adhesion to the urinary tract epithelium. A 2020 meta-analysis, which included eight studies, ultimately analyzed data from only 163 patients. While the results are promising, additional research is required to establish the optimal dosage and treatment duration for D-mannose [101].

### 5.3. Nanotechnology-Based Diagnosis of UTIs

UTIs represent one of the most prevalent bacterial infections, impacting approximately 50% of individuals at least once in their lifetime. In the absence of timely diagnosis and appropriate intervention, UTIs possess the potential to result in severe health ramifications [102]. Furthermore, the development of a swift, early, and dependable methodology for the detection of uropathogens could prove to be indispensable during clinical evaluations. Nanoscience constitutes a highly sophisticated field that has garnered significant scientific interest due to its myriad of potential applications within the biomedical domain [103]. To mitigate the challenges associated with conventional diagnostic methodologies, the integration of nanomedicine with these techniques may enhance the management of UTIs. Nanostructures exhibit superior efficacy in minimizing toxicity and resistance while concurrently reducing the costs associated with traditional diagnostic methods [104].

As previously indicated, the presently utilized techniques of urinalysis are intricate and protracted, and they exhibit deficiencies in both accuracy and precision. In light of their rapid response capabilities and enhanced precision and accuracy, photoluminescence (PL)-based biosensors have garnered increased scholarly attention in recent years [105]. In this context, Vasudevan et al. engineered a nanosensor employing cysteamine-conjugated ZnO nanoparticles (ZnO-Cys) for the detection of N-acyl-homoserine lactones (AHLs) produced by Gram-negative bacteria. AHLs are implicated in the modulation of pathogenicity in humans [106]. The synthesis of ZnO nanoparticles was accomplished through a microwave-assisted methodology, demonstrating commendable sensitivity (97%) and a linear detection range of 10–120 nM within artificial urine media. The efficacy of the nanosensor was validated through real-time analysis of AHLs synthesized by *Pseudomonas aeruginosa* (MCC3101), thereby corroborating their overall sensitivity and specificity [107].

In an endeavor to facilitate the early diagnosis of UTIs, a portable bacteria-capturing nanochip, predicated on surface-enhanced Raman scattering (SERS), was developed by Yang et al. for the direct detection of three species of uropathogens *(Proteus mirabilis* PRM1, *E. coli* CFT 073, and *Pseudomonas aeruginosa* PAO1) from urine samples. Initially, the chip was functionalized with NH3+ groups to enhance the electrostatic attraction of negatively charged bacteria. Following the capture of bacteria by the chip, silver nanoparticles (Ag NPs) were employed to obtain Raman fingerprint spectra of the bacterial specimens. The SERS-based chip successfully identified three species of UTI bacteria at low concentrations (10^5 cells/mL) through discriminant analysis (a chemometric approach). Moreover, the nanochip provided fingerprint data of the bacteria directly from artificial urine and Luria–Bertani (LB) culture media without necessitating any pre-treatment of the samples [108].

In a separate investigation, Alhogail et al. engineered a swift, adaptive, and highly accurate colorimetric nanosensor utilizing magnetic nanoparticles (MNPs) for the in vivo detection of *Pseudomonas aeruginosa* [109]. The nanoplatform was predicated on a specific protease substrate analysis of the proteolytic activity exhibited by *Pseudomonas aeruginosa*. The substrate was covalently bonded to MNPs at its N-terminus and linked to a gold (Au) sensor surface at its C-terminus. Generally, the Au nanosensor presents a black appearance to the unaided observer due to its MNP coating; however, upon proteolytic activity, the peptide–MNP complexes are cleaved and subsequently attracted by an external magnet. Consequently, the sensor surface transitions to a visible golden hue observable to the naked eye. In vitro, the biosensor successfully detected the presence of *Pseudomonas aeruginosa* at a concentration of 102 colony-forming units (cfu)/mL in under 1 min, while also demonstrating efficacy in identifying *P. aeruginosa* in clinical samples obtained from patients [109,110,111].

In environments characterized by limited resources, the point-of-care detection of pathogens within biological specimens necessitates a methodology that is rapid, straightforward, economical, compact, and precise [112]. Schecter et al. devised a bespoke fidget spinner incorporating gold nanoparticles (Au NPs) that facilitates the rapid concentration of pathogens by over 100-fold in 1 mL samples of undiluted urine, aimed at enabling the on-device colorimetric assessment of bacterial loads and pathogen identification [113]. This system enabled the on-site, unaided eye detection of infections in urine samples sourced from 39 patients suspected of having urinary tract infections (UTIs) within a timeframe of 50 min. Furthermore, the authors illustrated that the system could be operationalized for 30 clinical UTI samples to execute an antimicrobial susceptibility analysis for the antimicrobial agents ciprofloxacin and cefazolin within 120 min. This innovative fidget spinner could serve as a cost-effective portable apparatus for the swift concentration and identification of pathogens in urine samples within resource-constrained settings [114].

A mobile origami sensor with the capability of detecting UTIs attributable to *E. coli* in less than 7 min was devised by Adrover-Jaume et al. [115]. This sensor was constructed from a single piece of paper embedded with antibody-decorated Au NPs, forming an origami nanosensor. Upon exposure to urine samples containing *E. coli*, the biosensors generated colored spots on the paper strip, which could be quantified in real-time via a mobile application through pixel analysis. The examinations exhibited a high degree of precision and did not cross-react with other uropathogenic organisms [80]. Moreover, when assessed using a panel of 57 urine samples derived from patients, the biosensors produced only one false negative result, thereby demonstrating robust specificity and sensitivity. This outcome, coupled with rapid assessment times and smartphone-enabled identification, positions such biosensors as a valuable system for the accurate detection of UTIs [86].

Electrochemical nanosensors are particularly well suited for urinary diagnostics due to their remarkable efficiency, affordability, and capability to detect a diverse array of target molecules, including nucleic acids and protein biomarkers [116]. To facilitate the expeditious measurement of the urinary tract infection (UTI) lactoferrin biomarker, Pan et al. devised an electrochemical immunosensor (comprising a nanoarray of self-assembled monolayer alkanethiolates) utilizing serum samples from infected individuals [117]. Lactoferrin serves as a biomarker for pyuria, indicative of white blood cell presence in urine, which is a critical symptom of UTIs [118]. The limit of detection (LOD) established in this study was 145 pg/mL, and the concurrent identification of bacterial nucleic acid (specifically 16S rRNA) alongside host immune response-related protein (lactoferrin) on a singular sensor array demonstrated the capability for the multichannel detection of both uropathogens and lactoferrin. These results established the inaugural interconnected nanoplatform for simultaneous quantitative pathogen detection and assessment of host immune responses [116,117,118,119,120,121].

The commercially available colorimetric urine dipstick for early UTI detection presents certain limitations. The identification and quantification of urinary leukocyte esterase (LE) remain ambiguous concerning their predictive value for UTIs [119]. To address this issue, Ho et al. proposed a paper-based analytical device (PAD) for LE detection (designated as LE-PAD) as a proof-of-concept for a quantitative UTI diagnostic test [120]. The LE-PAD comprises a composite coating of 3-(N-tosyl-L-alaninyloxy)-5-phenylpyrrole (PE) and 1-diazo-2-naphthol-4-sulfonic acid (DAS) deposited on a Ag film or silver nanoparticles (Ag NPs). The analysis revealed that the quantity of LE quantified via LE-PADs was indicative of UTI diagnosis, yielding an area of 0.875 (95% confidence interval, 0.704–1.000) under the receiver operating characteristic curve. Utilizing an acceptable cut-off value, the diagnostic specificity and sensitivity of the LE-PAD were determined to be 87.5% and 92.3%, respectively, in contrast to urine dipstick LE positivity rates of 62.5% and 76.9%, respectively. The LE-PAD exhibited positive and negative likelihood ratios of 11.38 and 0.14, respectively, for UTI diagnosis, suggesting that this innovative LE-PAD may constitute a valid diagnostic approach [99,113].

Recent advancements have led to the development of novel biosensors predicated on the unique plasmonic energy transfer properties of nanometallic crossed surface-relief gratings (CSRGs) [114]. However, CSRG-based nanosensing has been predominantly confined to spectroscopic methodologies, failing to leverage its potential for integration with ubiquitous electronic devices [121]. Wang et al. introduced an innovative nanosensor incorporating surface plasmon resonance imaging (SPRi) facilitated by CSRGs. The imaging system employed two-dimensional nanoplasmonic gratings to enable a specific transfer of plasmonic energy among metallic nanostructures [121]. Finite-difference time-domain (FDTD) simulations demonstrated that, owing to plasmon resonance occurring at the metal–dielectric interface, CSRG-enabled SPRi was associated with an electric field intensity enhancement of approximately 30-fold. The system’s capacity for the rapid (<35 min) and label-free detection of uropathogenic *E coli* in phosphate-buffered saline (PBS) and human urine samples, ranging from 10^3^ to 10^9^ colony-forming units per milliliter (cfu/mL), underscored its potential for biomedical applications. The platform’s LOD was approximately 100 cfu/mL, which is three orders of magnitude lower than the clinical LOD for UTI detection. The sensing capability of the platform was experimentally validated by diagnosing variations in the bulk refractive index (RI), achieving a sensitivity of 382.2 nm/RI units (RIU) and a resolution of 10^−6^ RIU [122].

## 6. International Recommendations

According to the American Urological Association (AUA), Canadian Urological Association (CUA), and Society of Urodynamics, Female Pelvic Medicine, most recommendations for UTI diagnosis and treatment are categorized as level B or C. Recurring UTI diagnosis should always be confirmed by urine culture, and before starting treatment, the urinalysis and culture results must be reviewed. If symptoms are severe, antibiotics may be initiated before lab results [78,79,80]. Asymptomatic bacteriuria does not require treatment or testing. The antibiotic treatment of symptomatic UTIs should be based on antibiogram results, using nitrofurantoin, trimethoprim-sulfamethoxazole, or fosfomycin, typically for no more than seven days, with parenteral administration when needed. If symptoms are resolved, post-treatment tests are not necessary, but if they persist, a repeat culture should guide further treatment. Vaginal estrogen therapy is recommended for post- and perimenopausal women without contraindications. Prophylactic cranberry use and alternative therapies are supported in order to avoid antibiotic resistance, in line with WHO recommendations [113].

The European Association of Urology (EAU)’s guidelines include an updated literature review and recommendations for strength levels. They advise against diagnosing or treating asymptomatic bacteriuria, except for in pregnant women or those with bladder membrane damage [118]. Uncomplicated UTI diagnosis should rely on clinical symptoms unless vaginal infection is suspected, with urinary culture recommended for acute pyelonephritis, pregnancy, or unusual or persistent symptoms. Fosfomycin, pivampicillin, or nitrofurantoin are first-line treatments, while aminopenicillins and fluoroquinolones are not recommended for uncomplicated cystitis. Recurring UTIs should be confirmed with a culture and antibiogram, and non-antibiotic prevention should prioritize behavioral interventions and immune system stimulation with OM-89. Antibiotic prophylaxis is recommended only if non-antibiotic measures fail, with short-term treatments following successful interventions [123].

Postmenopausal women should receive estrogen therapy and behavioral modifications. Imaging use in uncomplicated cystitis is weakly supported, but urinalysis, urine culture, and imaging are recommended for pyelonephritis. Short courses of fluoroquinolones are advised for uncomplicated pyelonephritis, with hospitalization for intravenous antibiotics only when necessary [116]. Nitrofurantoin, fosfomycin, and pivampicillin are not recommended for pyelonephritis, and for complicated cases, aminoglycosides with amoxicillin or second-generation cephalosporins are suggested, or intravenous third-generation cephalosporins if generalized symptoms appear. Ciprofloxacin is recommended for non-hospitalized patients who have not used fluoroquinolones recently; however, prior use within the last six months is a contraindication. Routine antibiotic treatment after catheter removal is not advised [124].

The National Institute for Health and Care Excellence (NICE)’s 2020 guidelines recommend a 3-day course for treating lower UTIs outside of pregnancy, with treatment lasting from 3 to 6 days for older women. Nitrofurantoin or trimethoprim is recommended first-line, with fosfomycin or pivampicillin as second-line options. Pregnant women should receive 7-day treatments, ensuring no recent antibiotic resistance to the chosen drug. Asymptomatic bacteriuria should be treated with nitrofurantoin, amoxicillin, or cefalexin, while symptomatic infections should be treated with amoxicillin or cephalexin [90].

## 7. Conclusions

UTIs are one of the most prevalent infectious diseases affecting women across all age groups, with the highest incidence observed among pregnant and postmenopausal women. The pathogenesis of UTIs is often linked to pathogens originating from the gastrointestinal tract, which ascend through the urethra to infect the urinary system. Due to the potential for complications, a timely diagnosis and appropriate treatment are crucial.

The management of UTIs requires a balanced approach to antibiotic use, as excessive or inappropriate antibiotic administration can contribute to the development of antibiotic-resistant bacterial strains. In cases of asymptomatic bacteriuria, antibiotic therapy is not always warranted, particularly if the patient does not exhibit symptoms. The current clinical guidelines emphasize the importance of distinguishing asymptomatic bacteriuria from symptomatic infections, as the indiscriminate use of antibiotics increases the risk of resistance. Alternative strategies are recommended, especially for managing chronic infections and asymptomatic bacteriuria, where the goal is to prevent recurrence using non-antibiotic interventions.

In the context of recurrent UTIs, non-antibiotic preventive strategies are emphasized as the first line of intervention. These strategies prioritize behavioral modifications and the modulation of immune system responses to reduce recurrence rates. Professional organizations such as the AUA, CUA, and SUFU recommend a comprehensive review of urinalysis and culture results prior to initiating treatment. For postmenopausal and perimenopausal women, the use of vaginal estrogen therapy is advised in the absence of contraindications.

Similarly, the EAU underscores the importance of confirming the diagnosis of recurrent UTIs through urine culture. Non-antibiotic preventive measures are given precedence, with immune stimulation using OM-89 (an extract of *E. coli*) highlighted as a promising approach. Other evidence-based strategies include the use of prophylactic cranberry products, which have been shown to reduce UTI recurrence and limit the need for antibiotics, thereby mitigating the risk of developing antibiotic-resistant pathogens.

This shift toward non-antibiotic treatment and prevention is driven by the growing global threat of antimicrobial resistance. Clinical guidelines from leading urological and gynecological associations aim to promote rational antibiotic use, encourage the adoption of evidence-based non-antibiotic therapies, and support the implementation of behavioral interventions to reduce UTI recurrence. These recommendations underscore the importance of patient-centered care, with an emphasis on personalized treatment plans that prioritize both efficacy and antimicrobial stewardship.

## Figures and Tables

**Table 2:** Risk factors and prevalence of UTIs in different groups [77,78,79,80,81,82].

Groups	Risk Factors	Prevalence	Key Findings
Peri- and postmenopausal women	Estrogen deficiency, urinary incontinence, vaginal atrophy	4–19% (asymptomatic)	Topical estrogen reduces UTI risk (OR = 0.3)
Women with type 2 diabetes	Poor glycemic control, disease duration	17.5%	Diabetes doubles UTI risk (OR = 2.2), asymptomatic bacteriuria
Women with epilepsy	Use of AEDs (phenytoin, carbamazepine, etc.)	58% (requiring treatment)	AEDs increase UTI risk (ORs up to 1.78 for phenytoin)
Pregnant women	Urine pH changes, diabetes, proteinuria	2–8% (asymptomatic), 50–60% (overall)	UTIs linked to preterm birth and other complications
Patients with indwelling catheters	Catheterization, antiseptic use	15.4–86.6% (recurrent)	Antibiotic prophylaxis reduces risk of symptomatic UTI

**Table 3:** The recommended dosage of antibiotics is formulated based on the protocols established by multiple urological associations (EAU, SSGO, AWMF, AUA/CUA/SUFU, and KASRH) [55,84,85].

Indication	Antibiotics	Dosage	Treatment Duration	Key Findings
Prophylaxis in asymptomatic bacteriuria (Non-Pregnant)	Trimethoprim	100 mg 1× a day	Continuous	Contraindicated in pregnancy; folic acid antagonist
	Nitrofurantoin	50–100 mg a day	Continuous	Avoid in late pregnancy (risk of hemolytic anemia)
	Cephalexin	125–250 mg 1× a day	Continuous	Safe during pregnancy
	Fosfomycin	3 g every 10 days	Continuous	Safe in pregnancy and non-pregnant state
	Trimethoprim + Sulfamethoxazole	40/200 mg 1× a day	Continuous	Contraindicated in first trimester (neural tube defects)
Prophylaxis in asymptomatic bacteriuria (Pregnant)	Cephalexin	250 mg	Continuous	Preferred in pregnancy
	Nitrofurantoin	50–100 mg	Continuous	Avoid after 37 weeks of pregnancy
	Fosfomycin	3 g every 10 days	Continuous	Safe for use in pregnancy
Uncomplicated cystitis (Non-Pregnant)	Fosfomycin	3 g	1 day	Safe for single-dose therapy
	Nitrofurantoin	50–100 mg a day	5 days	Safe in early pregnancy but avoid in third trimester
	Extended-release Nitrofurantoin	100 mg 2× a day	5 days	Avoid near delivery
	Trimethoprim + Sulfamethoxazole	80/400 mg 2× a day	3 days	Avoid in pregnancy; folic acid antagonist
	Trimethoprim	100 mg 2× a day	3–5 days	Avoid in pregnancy
	Cephalosporins (e.g., Cefuroxime)	500 mg 2× a day	3 days	Safe for use during pregnancy
	Pivampicillin	400 mg 3× a day	3–5 days	Safe for pregnant women
Uncomplicated cystitis (Pregnant)	Cephalexin	500 mg 2× a day	3 days	First-line option for pregnant women
	Fosfomycin	3 g	1 day	Safe for pregnancy, short duration
	Nitrofurantoin	50–100 mg a day	5 days	Avoid in late pregnancy
Complicated cystitis (Non-Pregnant)	Ciprofloxacin	500–750 mg 2× a day	5 days	Contraindicated in pregnancy
	Levofloxacin	750 mg a day	5 days	Contraindicated in pregnancy
Complicated cystitis (Pregnant)	Cephalexin	500 mg 2× a day	7–10 days	Safe and effective in pregnancy
Pyelonephritis, parenteral therapy first line of treatment (Non-Pregnant)	Levofloxacin	750 mg a day	7–14 days	Fluoroquinolones contraindicated in pregnancy
	Ciprofloxacin	400 mg 2× a day	7–14 days	Avoid during pregnancy
	Cefotaxime	2 g 3× a day	7–14 days	Safe in pregnancy
	Ceftriaxone	1–2 g a day	7–14 days	Safe in pregnancy, widely used
Pyelonephritis, parenteral therapy first line of treatment (Pregnant)	Ceftriaxone	1–2 g a day	7–14 days	First-line option during pregnancy
	Cefotaxime	1–2 g 2× a day	7–14 days	Safe in pregnancy
Pyelonephritis, parenteral therapy second line of treatment (Non-Pregnant)	Cefepime	1–2 g 2× a day	7–14 days	Safe in pregnancy
	Piperacillin/tazobactam	2.5–4.5 g 3× a day	7–14 days	Safe in pregnancy
	Gentamycin	5 mg/kg a day	7–14 days	Use with caution due to ototoxicity risk
	Amikacin	15 mg/kg a day	7–14 days	Use with caution in pregnancy

## References

[1] Foxman B., Brown P. (2003). Epidemiology of urinary tract infections: Transmission and risk factors, incidence, and costs. Infect. Dis. Clin. N. Am..

[2] Butler C.C., Hawking M.K.D., Quigley A., McNulty C.A.M. (2015). Incidence, severity, help seeking, and management of uncomplicated urinary tract infection: A population-based survey. Br. J. Gen. Pract..

[3] Grobeisen-Duque O., Mora-Vargas C.D., Aguilera-Arreola M.G., Helguera-Repetto A.C. (2023). Cycle Biodynamics of Women’s Microbiome in the Urinary and Reproductive Systems. J. Clin. Med..

[4] Mancuso G., Midiri A., Gerace E., Marra M., Zummo S., Biondo C. (2023). Urinary Tract Infections: The Current Scenario and Future Prospects. Pathogens.

[5] Sanyaolu L.N., Hayes C.V., Lecky D.M., Ahmed H., Cannings-John R., Weightman A., Edwards A., Wood F. (2023). Patients’ and Healthcare Professionals’ Experiences and Views of Recurrent Urinary Tract Infections in Women: Qualitative Evidence Synthesis and Meta-Ethnography. Antibiotics.

[6] Ikahelmo R., Siitonen A., Heiskanen T., Karkkainen U., Kuosmanen P., Lipponen P., Mäkelä P.H. (1996). Recurrence of Urinary Tract Infection in a Primary Care Setting: Analysis of a I-Year Follow-up of 179 Women. Clin. Infect. Dis..

[7] Mititelu M., Olteanu G., Neacșu S.M., Stoicescu I., Dumitrescu D.-E., Gheorghe E., Tarcea M., Busnatu Ș.S., Ioniță-Mîndrican C.-B., Tafuni O. (2024). Incidence of Urinary Infections and Behavioral Risk Factors. Nutrients.

[8] Zeng Z., Zhan J., Zhang K., Chen H., Cheng S. (2022). Global, Regional, and National Burden of Urinary Tract Infections from 1990 to 2019: An Analysis of the Global Burden of Disease Study 2019. World J. Urol..

[9] Storme O., Tiran Saucedo J., Garcia-Mora A., Dehesa-Davila M., Naber K.G. (2019). Risk factors and predisposing conditions for urinary tract infection. Ther. Adv. Urol..

[10] Bader M.S., Loeb M., Brooks A.A. (2017). An update on the management of urinary tract infections in the era of antimicrobial resistance. Postgrad. Med..

[11] Olin S.J., Bartges J.W. (2015). Urinary tract infections: Treatment/comparative therapeutics. Vet. Clin. N. Am. Small Anim. Pract..

[12] Paduch D.A. (2007). Viral lower urinary tract infections. Curr. Urol. Rep..

[13] Chuang F.C., Kuo H.C. (2013). Increased urothelial cell apoptosis and chronic inflammation are associated with recurrent urinary tract infection in women. PLoS ONE.

[14] Semins M.J., Shore A.D., Makary M.A., Weiner J., Matlaga B.R. (2012). The impact of obesity on urinary tract infection risk. Urology.

[15] Tavakol Z., Ghannadi S., Tabesh M.R., Halabchi F., Noormohammadpour P., Akbarpour S., Alizadeh Z., Nezhad M.H., Reyhan S.K. (2023). Relationship between physical activity, healthy lifestyle and COVID-19 disease severity; a cross-sectional study. Z. Gesundh. Wiss..

[16] Eells S.J., Bharadwa K., McKinnell J.A., Miller L.G. (2014). Recurrent urinary tract infections among women: Comparative effectiveness of 5 prevention and management strategies using a Markov chain Monte Carlo model. Clin Infect Dis..

[17] Kong L.C., Holmes B.A., Cotillard A., Habi-Rachedi F., Brazeilles R., Gougis S., Gausserès N., Cani P.D., Fellahi S., Bastard J.P. (2014). Dietary patterns differently associate with inflammation and gut microbiota in overweight and obese subjects. PLoS ONE.

[18] Carpenter R.E. (2024). Beyond standard urine culture: Advanced molecular testing for urinary tract infections. SAR J. Med..

[19] Mestrovic T., Matijasic M., Peric M., Cipcic Paljetak H., Baresic A., Verbanac D. (2020). The Role of Gut, Vaginal, and Urinary Microbiome in Urinary Tract Infections: From Bench to Bedside. Diagnostics.

[20] Sannes M.R., Kuskowski M.A., Owens K., Gajewski A., Johnson J.R. (2004). Virulence Factor Profiles and Phylogenetic Background of *Escherichia coli* Isolates from Veterans with Bacteremia and Uninfected Control Subjects. J. Infect. Dis..

[21] Chen Y.Y., Su T.H., Lau H.H. (2021). Estrogen for the prevention of recurrent urinary tract infections in postmenopausal women: A meta-analysis of randomized controlled trials. Int. Urogynecol. J..

[22] Mtshali A., San J.E., Osman F., Garrett N., Balle C., Giandhari J., Onywera H., Mngomezulu K., Mzobe G., de Oliveira T. (2021). Temporal Changes in Vaginal Microbiota and Genital Tract Cytokines Among South African Women Treated for Bacterial Vaginosis. Front. Immunol..

[23] Brady S.S., Berry A., Camenga D.R., Fitzgerald C.M., Gahagan S., Hardacker C.T., Harlow B.L., Hebert-Beirne J., LaCoursiere D.Y., Lewis J.B. (2020). Applying concepts of life course theory and life course epidemiology to the study of bladder health and lower urinary tract symptoms among girls and women. Neurourol. Urodyn..

[24] Pat J.J., Witte L.P.W., Steffens M.G., Vernooij R.W.M., Marcelissen T.A.T., Fuentes P., Garcia-Perdomo H.A., Pardo-Hernandez H., Blanker M.H. (2022). Quality appraisal of clinical guidelines for recurrent urinary tract infections using AGREE II: A systematic review. Int. Urogynecol. J..

[25] Hilt E.E., Parnell L.K., Wang D., Stapleton A.E., Lukacz E.S. (2023). Microbial Threshold Guidelines for UTI Diagnosis: A Scoping Systematic Review. Pathol. Lab. Med. Int..

[26] Mandell L.A., Wunderink R.G., Anzueto A., Bartlett J.G., Campbell G.D., Dean N.C., Dowell S.F., File T.M., Musher D.M., Niederman M.S. (2007). Infectious Diseases Society of America/American Thoracic Society Consensus Guidelines on the Management of Community-Acquired Pneumonia in Adults. Clin. Infect. Dis..

[27] Wagenlehner F., Nicolle L., Bartoletti R., Gales A.C., Grigoryan L., Huang H., Hooton T., Lopardo G., Naber K., Poojary A. (2022). A global perspective on improving patient care in uncomplicated urinary tract infection: Expert consensus and practical guidance. J. Glob. Antimicrob. Resist..

[28] Gonzalez G., Vaculik K., Khalil C., Zektser Y., Arnold C., Almario C.V., Spiegel B., Anger J. (2022). Using Large-scale Social Media Analytics to Understand Patient Perspectives about Urinary Tract Infections: Thematic Analysis. J. Med. Internet Res..

[29] World Health Organization (2000). Guidelines for the Collection of Clinical Specimens During Field Investigation of Outbreaks.

[30] Chibelean C.B., Petca R.C., Mareș C., Popescu R.I., Enikő B., Mehedințu C., Petca A. (2020). A clinical perspective on the antimicrobial resistance spectrum of uropathogens in a Romanian male population. Microorganisms.

[31] Grigoryan L., Mulgirigama A., Powell M., Schmiemann G. (2022). The emotional impact of urinary tract infections in women: A qualitative analysis. BMC Womens Health.

[32] Rando E., Giovannenze F., Murri R., Sacco E. (2022). A review of recent advances in the treatment of adults with complicated urinary tract infection. Expert Rev. Clin. Pharmacol..

[33] Abraham S.N., Miao Y. (2015). The nature of immune responses to urinary tract infections. Nat. Rev. Immunol..

[34] Chaban B., Links M.G., Jayaprakash T.P., Wagner E.C., Bourque D.K., Lohn Z., Albert A.Y., van Schalkwyk J., Reid G., Hemmingsen S.M. (2014). Characterization of the vaginal microbiota of healthy Canadian women through the menstrual cycle. Microbiome.

[35] Aagaard K., Riehle K., Ma J., Segata N., Mistretta T.A., Coarfa C., Raza S., Rosenbaum S., Van den Veyver I., Milosavljevic A. (2012). A metagenomic approach to characterization of the vaginal microbiome signature in pregnancy. PLoS ONE.

[36] Shrestha L.B., Baral R., Khanal B. (2019). Comparative study of antimicrobial resistance and biofilm formation among Gram-positive uropathogens isolated from community-acquired urinary tract infections and catheter-associated urinary tract infections. Infect. Drug Resist..

[37] van Buul L.W., Vreeken H.L., Bradley S.F., Crnich C.J., Drinka P.J., Geerlings S.E., Jump R.L.P., Mody L., Mylotte J.J., Loeb M. (2018). The Development of a Decision Tool for the Empiric Treatment of Suspected Urinary Tract Infection in Frail Older Adults: A Delphi Consensus Procedure. J. Am. Med. Dir. Assoc..

[38] Flores-Mireles A.L., Walker J.N., Caparon M., Hultgren S.J. (2015). Urinary tract infections: Epidemiology, mechanisms of infection and treatment options. Nat. Rev. Microbiol..

[39] Hannan T.J., Mysorekar I.U., Hung C.S., Isaacson-Schmid M.L., Hultgren S.J. (2010). Early severe inflammatory responses to uropathogenic E. coli predispose to chronic and recurrent urinary tract infection. PLoS Pathog..

[40] Maniam L., Vellasamy K.M., Ong T.A., Teh C.S.J., Jabar K.A., Mariappan V., Narayanan V., Vadivelu J., Pallath V. (2023). Genotypic characteristics of Uropathogenic *Escherichia coli* isolated from complicated urinary tract infection (cUTI) and asymptomatic bacteriuria-a relational analysis. PeerJ.

[41] Meister M.R., Wang C., Lowder J.L., Mysorekar I.U. (2021). Vaginal Estrogen Therapy Is Associated With Decreased Inflammatory Response in Postmenopausal Women With Recurrent Urinary Tract Infections. Female Pelvic Med. Reconstr. Surg..

[42] Whitehead A.L., Julious S.A., Cooper C.L., Campbell M.J. (2016). Estimating the sample size for a pilot randomised trial to minimise the overall trial sample size for the external pilot and main trial for a continuous outcome variable. Stat. Methods Med. Res..

[43] Cooper E., Jones L., Joseph A., Allison R., Gold N., Larcombe J., Moore P., McNulty C. (2020). Diagnosis and management of uti in primary care settings—A qualitative study to inform a diagnostic quick reference tool for women under 65 years. Antibiotics.

[44] Muhammad A., Khan S.N., Ali N., Rehman M.U., Ali I. (2020). Prevalence and antibiotic susceptibility pattern of uropathogens in outpatients at a tertiary care hospital. New Microbes New Infect..

[45] Teglbrænder-Bjergkvist S., Siersma V., Holm A. (2023). Severity and Bothersomeness of Urinary Tract Infection Symptoms in Women before and after Menopause. Antibiotics.

[46] Giesen L.G.M., Cousins G., Dimitrov B.D., van de Laar F.A., Fahey T. (2010). Predicting Acute Uncomplicated Urinary Tract Infection in Women: A Systematic Review of the Diagnostic Accuracy of Symptoms and Signs. BMC Fam. Pract..

[47] Baerheim A., Digranes A., Malterud K. (2003). Generalized Symptoms in Adult Women with Acute Uncomplicated Lower Urinary Tract Infection: An Observational Study. MedGenMed Medscape Gen. Med..

[48] Hoffmann T., Peiris R., Del Mar C., Cleo G., Glasziou P. (2020). Natural History of Uncomplicated Urinary Tract Infection without Antibiotics: A Systematic Review. Br. J. Gen. Pract..

[49] Little P., Turner S., Rumsby K., Warner G., Moore M., Lowes J.A., Smith H., Hawke C., Mullee M. (2006). Developing Clinical Rules to Predict Urinary Tract Infection in Primary Care Settings: Sensitivity and Specificity of near Patient Tests (Dipsticks) and Clinical Scores. Br. J. Gen. Pract..

[50] Gágyor I., Rentzsch K., Strube-Plaschke S., Himmel W. (2021). Psychometric Properties of a Self-Assessment Questionnaire Concerning Symptoms and Impairment in Urinary Tract Infections: The UTI-SIQ-8. BMJ Open.

[51] Holm A., Siersma V., Cordoba G.C. (2021). Diagnosis of Urinary Tract Infection Based on Symptoms: How Are Likelihood Ratios Affected by Age? A Diagnostic Accuracy Study. BMJ Open.

[52] Chegini Z., Khoshbayan A., Vesal S., Moradabadi A., Hashemi A., Shariati A. (2021). Bacteriophage therapy for inhibition of multi drug-resistant uropathogenic bacteria: A narrative review. Ann. Clin. Microbiol. Antimicrob..

[53] Drawz S.M., Bonomo R.A. (2010). Three decades of beta-lactamase inhibitors. Clin. Microbiol. Rev..

[54] Newlands A.F., Roberts L., Maxwell K., Kramer M., Price J.L., Finlay K.A. (2023). Development and psychometric validation of a patient-reported outcome measure of recurrent urinary tract infection impact: The Recurrent UTI Impact Questionnaire. Qual Life Res..

[55] Anger J., Lee U., Ackerman A.L., Chou R., Chughtai B., Clemens J.Q., Hickling D., Kapoor A., Kenton K.S., Kaufman M.R. (2019). Recurrent uncomplicated urinary tract infections in women: AUA/CUA/SUFU guideline. J. Urol..

[56] Aspevall O., Hallander H., Gant V., Kouri T. (2001). European Guidelines for Urinalysis: A Collaborative Document Produced by European Clinical Microbiologists and Clinical Chemists under ECLM in Collaboration with ESCMID. Clin. Microbiol. Infect..

[57] Cody J.D., Jacobs M.L., Richardson K., Moehrer B., Hextall A. (2012). Oestrogen therapy for urinary incontinence in post-menopausal women. Cochrane Database Syst. Rev..

[58] Sterne J.A.C., White I.R., Carlin J.B., Spratt M., Royston P., Kenward M.G., Wood A.M., Carpenter J.R. (2009). Multiple Imputation for Missing Data in Epidemiological and Clinical Research: Potential and Pitfalls. BMJ.

[59] Boyko E.J., Fihn S.D., Scholes D., Chen C.-L., Normand E.H., Yarbro P. (2002). Diabetes and the risk of acute urinary tract infection among postmenopausal women. Diabetes Care.

[60] Cobo T., Vergara A., Collado M.C., Herreros E., Bosch J., Sanchez-Garcia A.B., Lopez-Parellada R., Ponce J., Gratacos E. (2019). Characterization of vaginal microbiota in women with preterm labor with intra-amniotic inflammation. Sci. Rep..

[61] Wee B.A., Thomas M., Sweeney E.L., Frentiu F.D., Samios M., Ravel J., Gajer P., Myers G., Timms P., Allan J.A. (2018). A retrospective pilot study to determine whether the reproductive tract microbiota differs between women with a history of infertility and fertile women. Aust. N. Z. J. Obstet. Gynaecol..

[62] Haahr T., Jensen J.S., Thomsen L., Duus L., Rygaard K., Humaidan P. (2016). Abnormal vaginal microbiota may be associated with poor reproductive outcomes: A prospective study in IVF patients. Hum. Reprod..

[63] Price T.K., Hilt E.E., Thomas-White K., Mueller E.R., Wolfe A.J., Brubaker L. (2020). The urobiome of continent adult women: A cross-sectional study. BJOG.

[64] Moustafa A., Li W., Singh H., Moncera K.J., Torralba M.G., Yu Y., Manuel O., Biggs W., Venter J.C., Nelson K.E. (2018). Microbial metagenome of urinary tract infection. Sci. Rep..

[65] Govender Y., Gabriel I., Minassian V., Fichorova R. (2019). The Current Evidence on the Association Between the Urinary Microbiome and Urinary Incontinence in Women. Front. Cell. Infect. Microbiol..

[66] Lee D.S., Lee S.J., Choe H.S. (2018). Community-Acquired Urinary Tract Infection by *Escherichia coli* in the Era of Antibiotic Resistance. BioMed Res. Int..

[67] Baraka M.A., Hussain A.l., Lehaibi L.H., Al-Suwaidan H.N. (2021). Patterns of infections and antimicrobial drugs’ prescribing among pregnant women in Saudi Arabia: A cross sectional study. J. Pharm. Policy Pract..

[68] Bergbower S.B., Saad A.F., Williams-Bouyer N.M., Rajendran R. (2024). Implementation of an algorithm for testing, diagnosis, and antibiotic stewardship of asymptomatic bacteriuria in pregnancy. Am. J. Obstet. Gynecol. MFM.

[69] Betschart C., Albrich W.C., Brandner S., Faltin D., Kuhn A., Surbek D., Geissbühler V. (2020). Guideline of the Swiss Society of Gynaecology and Obstetrics (SSGO) on acute and recurrent urinary tract infections in women, including pregnancy. Schweiz. Med. Wochenschr..

[70] Kline K.A., Lewis A.L. (2016). Gram-Positive Uropathogens, Polymicrobial Urinary Tract Infection, and the Emerging Microbiota of the Urinary Tract. Microbiol. Spectr..

[71] Czajkowski K., Broś-Konopielko M., Teliga-Czajkowska J. (2021). Urinary tract infection in women. Prz. Menopauzalny.

[72] Song D., Shi Y. (2014). Immune system modifications and feto-maternal immune tolerance. Chin. Med. J..

[73] Liu X., Zhu L., Huang Z., Li Z., Duan R., Li H., Xie L., Chen X., Ding W., Chen B. (2022). A dynamic peripheral immune landscape during human pregnancy. Fundam. Res..

[74] Lee M., Bozzo P., Einarson A., Koren G. (2008). Urinary tract infections in pregnancy. Can. Fam. Physician.

[75] Immonen T., Jung E., Gallo D.M., Diaz-Primera R., Gotsch F., Whittaker P., Than N.G., Bosco M., Tarca A.L., Suksai M. (2023). Acute pyelonephritis in pregnancy and plasma syndecan-1: Evidence of glycocalyx involvement. J. Matern. Fetal Neonatal Med..

[76] Capobianco G., De Muro P., Lepedda A., Dessole M., Ambrosini G., Cherchi P.L., Formato M. (2014). Impact of first trimester fasting glycemic levels on expression of proteoglycans in pregnancy. J. Obstet. Gynaecol. Res..

[77] Kwok M., McGeorge S., Mayer-Coverdale J., Graves B., Paterson D.L., Harris P.N.A., Esler R., Dowling C., Britton S., Roberts M.J. (2022). Guideline of guidelines: Management of recurrent urinary tract infections in women. BJU Int..

[78] Naber K.G., Bonkat G., Wagenlehner F.M.E. (2020). The EAU and AUA/CUA/SUFU Guidelines on Recurrent Urinary Tract Infections: What is the Difference?. Eur. Urol..

[79] Zhang S., Li G., Qiao L., Lai D., He Z., An L., Xu P., Tiselius H.-G., Zeng G., Zheng J. (2022). The antibiotic strategies during percutaneous nephrolithotomy in China revealed the gap between the reality and the urological guidelines. BMC Urol..

[80] Kot B. (2019). Antibiotic Resistance Among Uropathogenic *Escherichia coli*. Pol. J. Microbiol..

[81] Prieto J.M., Schinella G.R. (2022). Anti-Inflammatory and Antioxidant Chinese Herbal Medicines: Links between Traditional Characters and the Skin Lipoperoxidation “Western” Model. Antioxidants.

[82] Flower A., Wang L.Q., Lewith G., Liu J.P., Li Q. (2015). Chinese herbal medicine for treating recurrent urinary tract infections in women. Cochrane Database Syst. Rev..

[83] Jent P., Berger J., Kuhn A., Trautner B.W., Atkinson A., Marschall J. (2022). Antibiotics for Preventing Recurrent Urinary Tract Infection: Systematic Review and Meta-analysis. Open Forum Infect. Dis..

[84] Koepke M., Cerone J., Bologna R. (2009). Application and comparison of the AUA and EAU current recommendations for antibiotic prophylaxis in the urologic patient undergoing office procedures. Therapy.

[85] Mrkobrada M., Ying I., Mokrycke S., Dresser G.K., Elsayed S., Bathini V., Boyce E., Luke P. (2015). CUA Guidelines on antibiotic prophylaxis for urologic procedures. CUAJ-Can. Urol. Assoc. J..

[86] Meng H.-H., Xu D., Wang Q., Liu L., Liu W., Wang J. (2023). Maintaining Immune Homeostasis with Coptis Chinensis Water Extract to Mitigate Sepsis Severity via Modulating Gut Microbiome and Metabolism. J. Pharm. Biomed. Anal..

[87] Luty R.S., Fadil A.G., Najm J.M., Abduljabbar H.H., Kashmar S.A.A. (2020). Uropathogens antibiotic susceptibility as an indicator for the empirical therapy used for urinary tract infections: A retrospective observational study. Iran. J. Microbiol..

[88] Gomila A., Carratalà J., Eliakim-Raz N., Shaw E., Wiegand I., Vallejo-Torres L., Gorostiza A., Vigo J.M., Morris S., Stoddart M. (2018). Risk factors and prognosis of complicated urinary tract infections caused by Pseudomonas aeruginosa in hospitalized patients: A retrospective multicenter cohort study. Infect. Drug Resist..

[89] Karibayeva I., Moiynbayeva S., Akhmetov V., Yerkenova S., Shaikova K., Moshkalova G., Mussayeva D., Tarakova B. (2024). Interrupted time series analysis of the impact of the COVID-19 pandemic and compulsory social health insurance system on fertility rates: A study of live births in Kazakhstan, 2019–2023. Front. Public Health.

[90] Yerkenova S., Lokshin V., Saduakassova S., Zhabchenko I., Damulina D., BayanImasheva A. (2023). Preconception care to improve pregnancy outcomes in COVID-19 survival Women: A systematic review. Res. J. Pharm. Technol..

[91] Engelsöy U., Svensson M.A., Demirel I. (2021). Estradiol Alters the Virulence Traits of Uropathogenic *Escherichia coli*. Front. Microbiol..

[92] Pendharkar S., Skafte-Holm A., Simsek G., Haahr T. (2023). Lactobacilli and Their Probiotic Effects in the Vagina of Reproductive Age Women. Microorganisms.

[93] Cherpes T.L., Meyn L.A., Krohn M.A., Lurie J.G., Hillier S.L. (2003). Association between Acquisition of Herpes Simplex Virus Type 2 in Women and Bacterial Vaginosis. Clin. Infect. Dis..

[94] Skafte-Holm A., Humaidan P., Bernabeu A., Lledo B., Jensen J.S., Haahr T. (2021). The Association between Vaginal Dysbiosis and Reproductive Outcomes in Sub-Fertile Women Undergoing IVF-Treatment: A Systematic PRISMA Review and Meta-Analysis. Pathogens.

[95] Lokken E.M., Manhart L.E., Kinuthia J., Hughes J.P., Jisuvei C., Mwinyikai K., Muller C.H., Mandaliya K., Jaoko W., McClelland R.S. (2021). Association between Bacterial Vaginosis and Fecundability in Kenyan Women Planning Pregnancies: A Prospective Preconception Cohort Study. Hum. Reprod..

[96] Leitich H., Kiss H. (2007). Asymptomatic Bacterial Vaginosis and Intermediate Flora as Risk Factors for Adverse Pregnancy Outcome. Best Pract. Res. Clin. Obstet. Gynaecol..

[97] Amabebe E., Anumba D.O.C. (2018). The Vaginal Microenvironment: The Physiologic Role of Lactobacilli. Front. Med..

[98] Zheng N., Guo R., Wang J., Zhou W., Ling Z. (2021). Contribution of Lactobacillus Iners to Vaginal Health and Diseases: A Systematic Review. Front. Cell. Infect. Microbiol..

[99] Yarbrough V.L., Winkle S., Herbst-Kralovetz M.M. (2015). Antimicrobial Peptides in the Female Reproductive Tract: A Critical Component of the Mucosal Immune Barrier with Physiological and Clinical Implications. Hum. Reprod. Update.

[100] Schwenger E.M., Tejani A.M., Loewen P.S. (2015). Probiotics for preventing urinary tract infections in adults and children. Cochrane Database Syst. Rev..

[101] Yazdanpour Z., Tadjrobehkar O., Shahkhah M. (2020). Significant association between genes encoding virulence factors with antibiotic resistance and phylogenetic groups in community acquired uropathogenic Escherichia coli isolates. BMC Microbiol..

[102] Ruiz Ramos J., Salavert Lleti M. (2019). Fosfomycin in infections caused by multidrug-resistant Gram-negative pathogens. Rev. Esp. Quimioter..

[103] Scangarella-Oman N.E., Hossain M., Hoover J.L., Perry C.R., Tiffany C., Barth A., Dumont E.F. (2022). Dose Selection for Phase III Clinical Evaluation of Gepotidacin (GSK2140944) in the Treatment of Uncomplicated Urinary Tract Infections. Antimicrob. Agents Chemother..

[104] Veeraraghavan B., Bakthavatchalam Y.D., Sahni R.D. (2021). Oral Antibiotics in Clinical Development for Community-Acquired Urinary Tract Infections. Infect. Dis. Ther..

[105] Vasudevan S., Srinivasan P., Rayappan J.B.B., Solomon A.P. (2020). A photoluminescence biosensor for the detection of N-acyl homoserine lactone using cysteamine functionalized ZnO nanoparticles for the early diagnosis of urinary tract infections. J. Mater. Chem. B.

[106] Gao H., Ye J., Zhao R., Zhan M., Yang G., Yu R. (2022). Pluripotency of endogenous AHL-mediated quorum sensing in adaptation and recovery of biological nitrogen removal system under ZnO nanoparticle long-term exposure. Sci. Total Environ..

[107] Yang D., Zhou H., Dina N.E., Haisch C. (2018). Portable bacteria-capturing chip for direct surface-enhanced Raman scattering identification of urinary tract infection pathogens. R. Soc. Open Sci..

[108] Alhogail S., Suaifan G.A., Bikker F.J., Kaman W.E., Weber K., Cialla-May D., Popp J., Zourob M.M. (2019). Rapid Colorimetric Detection of *Pseudomonas aeruginosa* in Clinical Isolates Using a Magnetic Nanoparticle Biosensor. ACS Omega.

[109] Cattoir V., Gilibert A., Le Glaunec J.-M., Launay N., Bait-Mérabet L., Legrand P. (2010). Rapid detection of Pseudomonas aeruginosa from positive blood cultures by quantitative PCR. Ann. Clin. Microbiol. Antimicrob..

[110] Lee C.S., Wetzel K., Buckley T., Wozniak D., Lee J. (2011). Rapid and sensitive detection of Pseudomonas aeruginosa in chlorinated water and aerosols targeting gyrB gene using real-time PCR. J. Appl. Microbiol..

[111] Yang J., Wang X., Sun Y., Chen B., Hu F., Guo C., Yang T. (2023). Recent Advances in Colorimetric Sensors Based on Gold Nanoparticles for Pathogen Detection. Biosensors.

[112] Schecter R.A., Shah J., Fruitman K., Milanaik R.L. (2017). Fidget spinners: Purported benefits, adverse effects and accepted alternatives. Curr. Opin. Pediatr..

[113] Mike L.A., Smith S.N., Sumner C.A., Eaton K.A., Mobley H.L. (2016). Siderophore vaccine conjugates protect against uropathogenic *Escherichia coli* urinary tract infection. Proc. Natl. Acad. Sci. USA.

[114] Adrover-Jaume C., Rojo-Molinero E., Clemente A., Russell S.M., Arranz J., Oliver A., de la Rica R. (2021). Mobile origami immunosensors for the rapid detection of urinary tract infections. Analyst.

[115] Duane S., Domegan C., Callan A., Galvin S., Cormican M., Bennett K., Murphy A., Vellinga A. (2016). Using qualitative insights to change practice: Exploring the culture of antibiotic prescribing and consumption for urinary tract infections. BMJ Open.

[116] Pan Y., Sonn G.A., Sin M.L., Mach K.E., Shih M.C., Gau V., Wong P.K., Liao J.C. (2010). Electrochemical immunosensor detection of urinary lactoferrin in clinical samples for urinary tract infection diagnosis. Biosens. Bioelectron..

[117] Griebling T.L. (2005). Urologic Diseases in America Project: Trends in Resource Use for Urinary Tract Infections in Men. J. Urol..

[118] Bustamante M., Oomah B.D., Oliveira W.P., Burgos-Díaz C., Rubilar M., Shene C. (2020). Probiotics and prebiotics potential for the care of skin, female urogenital tract, and respiratory tract. Folia Microbiol..

[119] Ho M.L., Liu W.F., Tseng H.Y., Yeh Y.T., Tseng W.T., Chou Y.Y., Huang X.R., Hsu H.C., Ho L.I., Pan S.W. (2020). Quantitative determination of leukocyte esterase with a paper-based device. RSC Adv..

[120] Wang D., Loo J.F.C., Chen J., Yam Y., Chen S.-C., He H., Kong S.K., Ho H.P. (2019). Recent Advances in Surface Plasmon Resonance Imaging Sensors. Sensors.

[121] Rafiee M., Chandra S., Ahmed H., McCormack S.J. (2021). Optimized 3D Finite-Difference-Time-Domain Algorithm to Model the Plasmonic Properties of Metal Nanoparticles with Near-Unity Accuracy. Chemosensors.

[122] Warzecha D., Pietrzak B., Urban A., Wielgoś M. (2021). How to avoid drug resistance during treatment and prevention of urinary tract infections. Prz. Menopauzalny.

[123] Gardiner B.J., Stewardson A.J., Abbott I.J., Peleg A.Y. (2019). Nitrofurantoin and fosfomycin for resistant urinary tract infections: Old drugs for emerging problems. Aust. Prescr..

[124] Kashouris E., Joseph A., Lewis T. (2023). Nitrofurantoin: What is the evidence for current UK guidance?. J. Antimicrob. Chemother..

